# XRCC1 and PCNA are loading platforms with distinct kinetic properties and different capacities to respond to multiple DNA lesions

**DOI:** 10.1186/1471-2199-8-81

**Published:** 2007-09-19

**Authors:** Oliver Mortusewicz, Heinrich Leonhardt

**Affiliations:** 1 Ludwig Maximilians University Munich, Department of Biology II, 82152 Planegg-Martinsried, Germany

## Abstract

**Background:**

Genome integrity is constantly challenged and requires the coordinated recruitment of multiple enzyme activities to ensure efficient repair of DNA lesions. We investigated the dynamics of XRCC1 and PCNA that act as molecular loading platforms and play a central role in this coordination.

**Results:**

Local DNA damage was introduced by laser microirradation and the recruitment of fluorescent XRCC1 and PCNA fusion proteins was monitored by live cell microscopy. We found an immediate and fast recruitment of XRCC1 preceding the slow and continuous recruitment of PCNA. Fluorescence bleaching experiments (FRAP and FLIP) revealed a stable association of PCNA with DNA repair sites, contrasting the high turnover of XRCC1. When cells were repeatedly challenged with multiple DNA lesions we observed a gradual depletion of the nuclear pool of PCNA, while XRCC1 dynamically redistributed even to lesions inflicted last.

**Conclusion:**

These results show that PCNA and XRCC1 have distinct kinetic properties with functional consequences for their capacity to respond to successive DNA damage events.

## Background

Mammalian cells have to deal with a wide variety of different DNA lesions caused by cellular metabolites, replication errors, spontaneous disintegration and environmental influences. These lesions can occur at successive times and in distant parts of the genome constituting a permanent threat to the genetic integrity. Numerous repair pathways have evolved to reestablish and maintain the genetic information [1,2]. The recent identification of DNA methyltransferase I at repair sites indicated that not only the genetic but also the epigenetic information is restored [3].

The repair of DNA lesions involves multiple steps including initial damage recognition, intracellular signaling and the recruitment of repair factors. For the latter step so called loading platforms are considered to play a central role by locally concentrating and coordinating repair factors at sites of DNA damage. These loading platforms have no enzymatic activity of their own but interact with numerous proteins through highly conserved binding motifs. XRCC1 (X-ray cross complementing factor 1) and PCNA (proliferating cell nuclear antigen) both fulfill these criteria and are therefore considered to act as central loading platforms in DNA replication and repair (reviewed in [4-6]).

XRCC1 was first identified in the Chinese Hamster ovary (CHO) mutant cell line EM9 [7]. This cell line shows a defect in single strand break repair (SSBR) and increased sensitivity to alkylating agents and ionizing irradiation resulting in elevated frequency of spontaneous chromosome aberrations and deletions. The importance of XRCC1 is further underlined by the embryonic lethality of *XRCC1*^-/- ^mice [8]. The fact that XRCC1 interacts with various proteins involved in SSBR and base excision repair (BER), including PARP-1, PARP-2 [9-11] Polymerase-β [12,13] and DNA Ligase III [9,14] suggests that XRCC1 acts as a loading platform in these pathways. Interestingly, XRCC1 also interacts with PCNA and it was proposed that this interaction facilitates efficient SSBR during DNA replication [15].

PCNA forms a homotrimeric ring around the DNA allowing both stable association with and sliding along the DNA double helix. Therefore PCNA is often referred to as a "sliding clamp" mediating interaction of various proteins with DNA in a sequence-independent manner. Photobleaching experiments have shown that in DNA replication PCNA acts as stationary loading platform for transiently interacting Okazaki fragment maturation proteins [16,17]. In the last few years it has become evident that PCNA is not only essential for DNA replication but also for various DNA repair pathways including nucleotide excision repair (NER) [18], base excision repair (BER) [19,20], mismatch repair (MMR) [21-23] and repair of double strand breaks (DSBs) [24,25]. Recently it has been shown, that accumulation of PCNA at DNA repair sites is independent of the RFC complex, which loads PCNA onto DNA during DNA replication [26]. Furthermore PCNA plays also an important role in postreplicative processes such as cytosine methylation and chromatin assembly [27,28]. In most cases, proteins involved in these processes directly bind to PCNA through a conserved PCNA-binding domain (PBD). This raises the question of how binding is coordinated and sterical hindrance avoided in DNA replication and repair. Recent studies have shown that posttranslational modifications of PCNA such as ubiquitinylation and sumoylation [29-34] mediate a switch between DNA replication and different repair pathways.

To study the dynamics of the two loading platforms XRCC1 and PCNA at DNA repair sites in Hela cells we used a combination of microirradiation, live cell microscopy and photobleaching techniques. We found that XRCC1 and PCNA exhibit distinct recruitment and binding kinetics at repair sites resulting in different capacities to respond to successive DNA damage events.

## Results and discussion

### XRCC1 is less tightly associated with repair sites than PCNA

**Figure 1:**
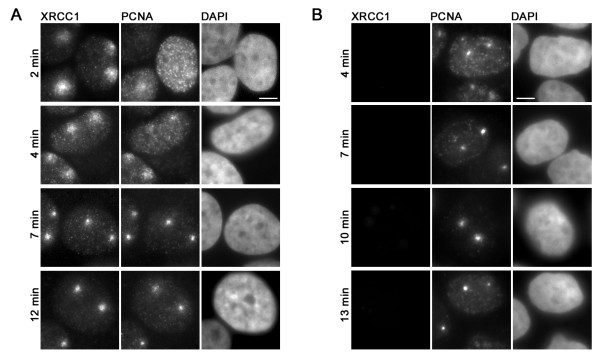
Immunochemical detection of endogenous XRCC1 and PCNA at DNA repair sites. Widefield fluorescence images of Hela cells are shown. Cells were fixed at indicated time points after laser microirradiation. (A) Both, XRCC1 and PCNA, accumulate at laser-induced DNA damage sites. (B) Microirradiated Hela cells were extracted with 0,5% Triton-X100 and 500 mM NaCl prior to fixation. After in situ extraction no endogenous XRCC1 can be detected at microirradiated sites while PCNA accumulations can still be observed. Scale bars, 5 μm.

XRCC1 and PCNA have no known enzymatic function, are present at repair sites and interact with a high number of different proteins suggesting that they act as loading platforms in DNA repair. To investigate the role of XRCC1 and PCNA in DNA repair we performed immunostainings of microirradiated Hela cells. We employed a confocal laser scanning microscope to generate DNA damage at preselected subnuclear sites with a long wavelength UV diode laser in BrdU-sensitized cells as described before [3,35]. Microirradiated sites stained positive for phosphorylated histone variant H2AX (γH2AX), a marker for double strand breaks (DSBs), and poly(ADP-Ribose) which is generated by PARP at single strand breaks (SSBs) (Additional file 1). This indicates that microirradiation with a 405 nm laser generates a mixture of different types of DNA damage that are substrates for distinct DNA repair pathways involving XRCC1 and/or PCNA. Immunofluorescence stainings with specific antibodies revealed that endogenous PCNA and XRCC1 are both present at DNA damage sites as early as 2–4 min after irradiation (Figure 1A). To investigate the binding properties of XRCC1 and PCNA at DNA repair sites we performed salt extraction experiments. Microirradiated cells were permeabilized for 30 s followed by extraction with phosphate buffer containing 500 mM NaCl for 1 min. Immediately after salt extraction, the cells were fixed and stained for endogenous proteins showing that XRCC1 and PCNA were both extracted in non-S phase cells that were not microirradiated. In microirradiated non-S phase cells only XRCC1 was extracted while PCNA could still be detected at DNA damage sites (Figure 1B), which is in good agreement with an earlier study, where detergent resistant foci of PCNA could be observed after local UV irradiation [36]. As previously reported [15] we also detected a partial colocalization of XRCC1 with PCNA at replication sites, but noticed dramatically different binding properties. Thus XRCC1 was readily extracted, whereas PCNA was still stably associated with sites of DNA replication (Figure 1B). Taken together these results show that endogenous XRCC1 and PCNA are both present at DNA replication and repair sites but exhibit different binding properties.

### Recruitment and mobility of XRCC1 and PCNA at DNA repair sites

To further investigate the dynamics detected with salt extraction experiments we combined the microirradiation technique with live cell microscopy and photobleaching analysis (FRAP). We first determined the recruitment kinetics of XRCC1 and PCNA in living cells by quantifying the amount of GFP- and RFP-tagged XRCC1 and PCNA accumulated at microirradiated sites. The intensity values were corrected for background and for total nuclear loss of fluorescence over the time course and normalized to the pre-irradiation value.

**Figure 2:**
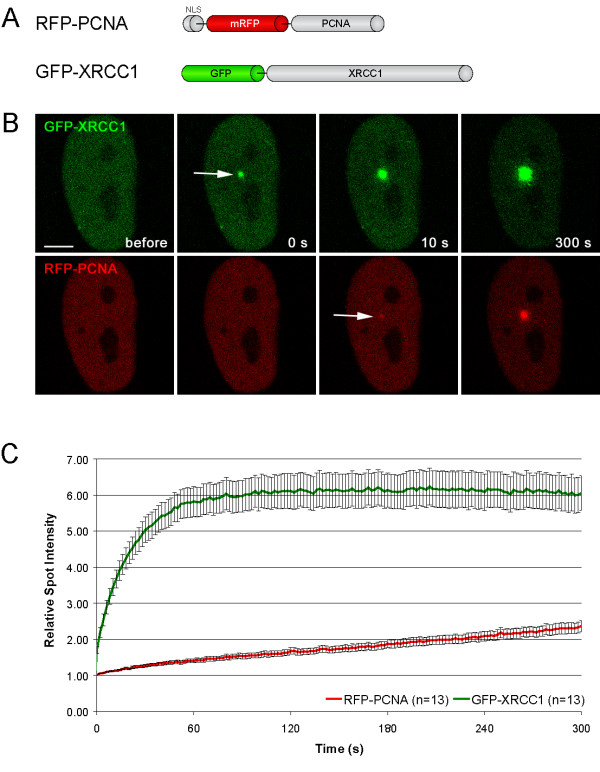
Recruitment of XRCC1 and PCNA at DNA damage sites in living cells. (A) Schematic representation of the fluorescent fusion proteins. (B) Live cell imaging of a microirradiated Hela cell coexpressing GFP-XRCC1 and RFP-PCNA. Accumulation of GFP-XRCC1 can be observed immediately after microirradiation, while RFP-PCNA accumulates with a short delay of about 2–10 s (indicated by arrows). (C) Quantitative evaluation of recruitment kinetics showing mean curves. Error bars represent the standard error of the mean. Immediate and fast recruitment of GFP-XRCC1 precedes slow and constant recruitment of RFP-PCNA at DNA damage sites. Scale bar, 5 μm.

A direct comparison of GFP- and RFP-tagged XRCC1 and PCNA showed a significantly slower recruitment of PCNA in contrast to the very fast accumulation of XRCC1 at microirradiated sites (Figure 2A). The fluorescence intensity of PCNA at the irradiated site increased during the observation period of 5 min, while XRCC1 accumulation reached a maximum about 1–2 min after irradiation (Figure 2B). These kinetic differences are in good agreement with earlier studies comparing the recruitment of XRCC1 and PCNA to laser-induced DNA damage sites [37].

**Figure 3:**
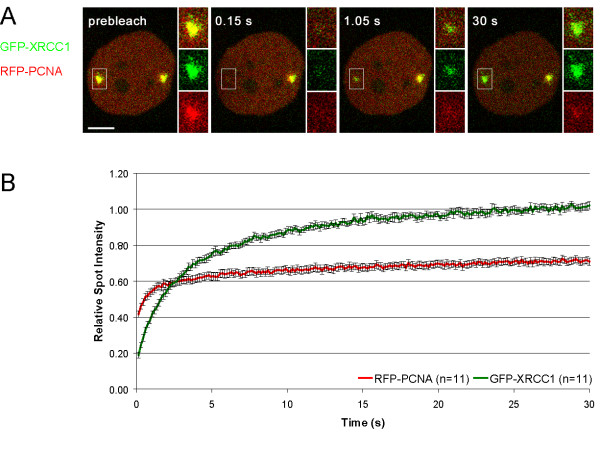
Mobility of XRCC1 and PCNA at DNA damage sites. (A) Two separate subnuclear spots of a transiently transfected Hela cell were microirradiated. The mobility of accumulated fluorescent fusion proteins was determined by bleaching one of the two spots 5 min after microirradiation and subsequent recovery measurements. Inset shows the bleached microirradiated site. Scale bar, 5 μm. (B) FRAP data from 11 individual experiments are shown as mean curves. Error bars represent the standard error of the mean.

Having shown that XRCC1 and PCNA are recruited with distinct kinetics we performed FRAP analysis to determine their dynamics at laser-induced DNA damage sites. Two separate spots were microirradiated in living cells coexpressing GFP-XRCC1 and RFP-PCNA. After 5 min one region was bleached with a high energy laser pulse for 300 ms and the fluorescence recovery was determined. After bleaching of the repair foci we observed complete recovery of the XRCC1 signal within 24 s (Figure 3). Since fluorescence intensity at repair sites had already peaked and did not increase any further, the measured recovery has to be attributed to a rapid turnover of XRCC1.

In contrast, no recovery of PCNA at DNA repair sites could be observed within the observation period, which is in good agreement with previous studies where DNA damage was induced by chemical agents or irradiation with a UV lamp [30,38].

**Figure 4:**
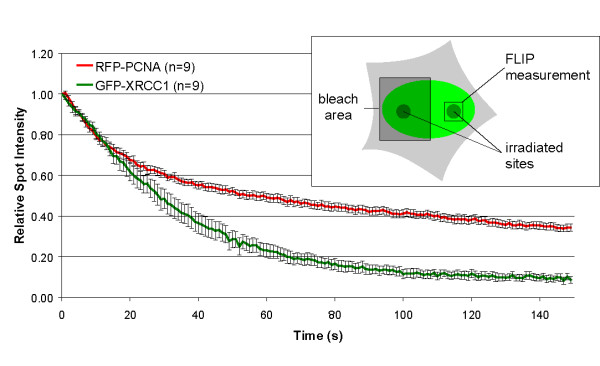
Different binding kinetics of XRCC1 and PCNA at DNA repair sites. FLIP data from 9 individual experiments are shown as mean curves. The scheme of the experiment is outlined in the inset. Two separate subnuclear spots of transiently transfected Hela cells were microirradiated. Half of the nucleus containing one irradiated site was repeatedly bleached for 1 s over a total time period of 150 s, starting 5 min after microirradiation. The decay of the fluorescence intensity at the microirradiated site within the non-bleached half of the nucleus was measured and plotted over time. Error bars represent the standard error of the mean.

To determine the dissociation kinetics of XRCC1 and PCNA from DNA damage sites we performed FLIP experiments in Hela cells expressing GFP-XRCC1 and RFP-PCNA. 5 min after microirradiation half of the nucleus was repeatedly bleached with a high energy laser pulse over a time period of 150 s and the loss of fluorescence at the microirradiated site located outside the bleaching area was determined (Figure 4, inset). The intensity values were corrected for background fluorescence and normalized to the pre-bleach value.

Within the first 10–15 s both fusion proteins showed a rapid loss of fluorescence due to depletion of highly mobile, unbound fluorescent molecules within the region of interest. After this initial phase XRCC1 and PCNA exhibited dramatically different dissociation kinetics. We could observe a rapid decrease of XRCC1 fluorescence to 10% of the initial intensity within the observation period while the intensity of PCNA was only reduced to 34% (Figure 4). This argues for a constant exchange of fluorescent XRCC1 molecules between the damage site and the bleached half of the nucleus, while most RFP-PCNA molecules remained bound at DNA repair sites.

These results show that the two loading platforms XRCC1 and PCNA exhibit distinct recruitment kinetics and mobility (association and dissociation rates) at DNA repair sites, which is consistent with an involvement of XRCC1 and PCNA in distinct repair pathways. On the one hand, PCNA is involved in repair pathways where the synthesis of long stretches of DNA requires a stable and processive repair machinery. On the other hand, XRCC1 is part of the short patch BER pathway where only a single nucleotide needs to be replaced and no processive and stable machinery is required.

To further investigate the role of XRCC1 and PCNA as central loading platforms in DNA repair we extended our photobleaching analysis to their respective interaction partners DNA Ligase III and I. In a previous study we compared the recruitment kinetics of theses highly conserved DNA Ligases and found that they are recruited to DNA repair sites with distinct kinetics. Using mutational analysis and binding studies we could show, that DNA Ligase I is recruited to repair sites through interaction with PCNA, while DNA Ligase III is recruited via its BRCT domain interacting with XRCC1 [35]. FRAP analysis revealed that both DNA Ligases show a high turnover at repair sites, with DNA Ligase I recovering faster than DNA Ligase III (Additional file 2). Interestingly, DNA Ligase III showed the same recovery rate as its loading platform XRCC1, while the mobility of DNA Ligase I and PCNA at repair sites differed dramatically (Additional file 2).

These results demonstrate that these loading platforms and their interacting repair factors have independent binding properties at repair sites. We speculate that even transient interaction of repair factors with their respective loading platform enhances the efficiency of DNA repair by local concentration of enzyme activities at repair sites, allowing faster recognition and binding of repair substrates.

### Flexible response of XRCC1 and PCNA to multiple DNA damage events

**Figure 5:**
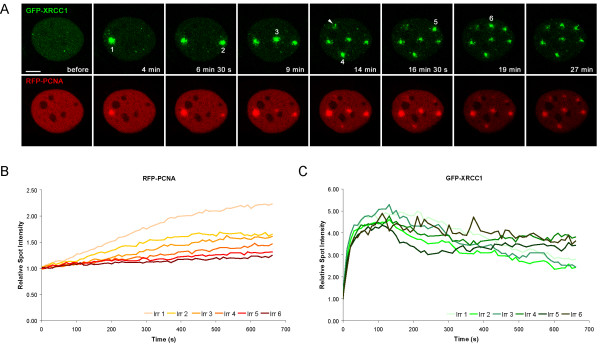
Flexible response of XRCC1 to multiple DNA damage events. (A) Consecutive/Successive DNA lesions were introduced with a time interval of 2.5 min, starting 4 min after microirradiation of the first spot. One spot irradiated in close proximity to the nucleoli was not evaluated (arrowhead) (B) The recruitment kinetics of XRCC1 and PCNA at consecutively microirradiated sites were evaluated and plotted over time. Representative curves of one Hela cell are shown.

To investigate whether the different binding properties of XRCC1 and PCNA have functional consequences we tested their ability to respond to multiple DNA lesions. Successive DNA lesions were introduced with a time interval of 2.5 min at separate spots and the recruitment kinetics were determined for each individual spot. We observed a constant decrease of PCNA accumulation at sites irradiated at later time points (Figure 5). In contrast, XRCC1 accumulation at early and late irradiated sites was similar.

These differences can be explained by the tight binding of PCNA at repair sites leading to a depletion of the cellular pool of PCNA molecules available for repair of subsequent damages.

In contrast, the dynamic binding of XRCC1 enables fast exchange between multiple DNA damages sites separated in time and space. Taken together these findings argue for a role of PCNA as a stationary loading platform in DNA repair allowing efficient and accurate repair, whereas the fast recruitment and high turnover of XRCC1 enables a flexible response to multiple DNA damage events occurring at distant sites and successive times in the genome.

## Conclusion

In summary, we found that XRCC1 and PCNA exhibit distinct recruitment and binding kinetics at repair sites, which goes beyond earlier studies comparing only the accumulation of XRCC1 and PCNA at repair sites [37]. Efficient repair of DNA lesions requires avid recognition of the damage and coordinated recruitment of a multitude of repair factors. The principle dilemma faced by the repair machinery is that the stable complex formation required for processivity and completion of multi-step processes is incompatible with a flexible response to later changes like subsequent DNA damages. Our live cell recruitment and photobleaching analyses showed that XRCC1 and PCNA represent opposite strategies. We clearly demonstrate that the stable binding of the processivity factor PCNA limits its capacity to respond to successive damage events. While the avid and transient binding of XRCC1 might be sufficient for single nucleotide replacement but allows a flexible response to multiple consecutive DNA lesions. This type of live cell analysis should also help to explore the flexibility of other repair factors and complex cellular machineries in response to changing requirements.

## Methods

### Cell culture and transfection

HeLa cells were cultured in DMEM containing 50 μg/ml gentamicin supplemented with 10% FCS. Cells grown on μ-slides (Ibidi) or on gridded coverslips were cotransfected with jetPEI (PolyPlus Transfection) or TransFectin transfection reagent (Bio-Rad) according to the manufacturers instructions. For microirradiation experiments cells were sensitized by incubation in medium containing BrdU (10 μg/ml) for 24–48 h.

### Expression plasmids

Mammalian expression constructs encoding translational fusions of human PCNA with either green (GFP) or red (RFP) fluorescent protein were previously described [17]. Red variants of the previously described GFP-Ligase III [3] and GFP-XRCC1 [39] were generated by replacing GFP with RFP [40] and termed RFP-Ligase III and RFP-XRCC1, respectively. In all cases expression was under the control of the CMV promoter. We tested all fusion proteins by expression in 293T cells followed by western blot analysis.

### Immunofluorescence and Detergent Extraction

Cells were fixed in 3,7% formaldehyde for 10 min and permeabilized with ice-cold methanol for 5 min. The following primary antibodies (diluted in PBS containing 2% BSA) were used: anti-γ H2AX (Ser139) rabbit antibody (Upstate), anti-PAR mouse monoclonal antibody (Trevigen), anti-XRCC1 mouse monoclonal antibody (Abcam) and anti-PCNA rat monoclonal antibody [41]. Secondary antibodies (diluted 1:400 in PBS containing 2% BSA) conjugated to Alexa Fluor 488, 555 or 647 (Molecular Probes) were used. Cells were counterstained with DAPI and mounted in Vectashield (Vector Laboratories). For in situ detergent extraction, cells were permeabilized for 30 s with 0,5% Triton X-100 in PBS and extracted for 1 min with 500 mM NaCl in PBS before fixation.

### Live-cell Microscopy, microirradiation and photobleaching experiments

Live cell imaging, mircorirradiation and photobleaching experiments were carried out with a Leica TCS SP2/AOBS confocal laser scanning microscope equipped with a UV-transmitting HCX PL 63×/1.4 oil objective. Fluorophores were exited using a 488 nm Ar laser line and a 561 nm diode laser line. The microscope was equipped with a heated environmental chamber set to 37°C. Confocal image series were typically recorded with a frame size of 256 × 256 pixels and a pixel size of 90 nm.

Microirradiation was carried out with a 405 nm diode laser set to maximum power at 100% transmission. Preselected spots of ~1 μm in diameter within the nucleus were microirradiated for 1 s. Before and after microirradiation confocal image series of one mid z-section were recorded at 2 s time interval (typically 6 pre-irradiation and 150 post-irradiation frames). For evaluation of recruitment kinetics, fluorescence intensities at the irradiated region were corrected for background and for total nuclear loss of fluorescence over the time course and normalized to the pre-irradiation value.

For FRAP analysis, a region of interest was selected and photobleached for 300 ms with all laser lines of the Ar-laser and the 561 nm DPSS laser set to maximum power at 100% transmission. Before and after bleaching, confocal image series were recorded at 150 ms time intervals (typically 10 prebleach and 200 postbleach frames). Mean fluorescence intensities of the bleached region were corrected for background and for total nuclear loss of fluorescence over the time course and normalized to the mean of the last 4 prebleach values.

For FLIP analysis, one half of the nucleus was repeatedly photobleached (typically 150 frames) with all laser lines of the Ar-laser and the 561 nm DPSS laser set to maximum power at 100% transmission for 1 s. Mean fluorescence intensities of the bleached region were corrected for background and normalized to the initial value.

For quantitative evaluation of microirradiation and photobleaching experiments, data of at least 9 nuclei were averaged and the mean curve as well as the standard error of the mean calculated and plotted using Microsoft Excel software.

Images of fixed cells were taken with a Zeiss Axiophot 2 widefield epifluorescence microscope using a Zeiss Plan-Apochromat 63×/1.40 oil objective and a cooled CCD camera (Visitron Systems).

## Abbreviations

BER: base excision repair

DSBs: double strand breaks

FLIP: fluorescence loss in photobleaching

FRAP: fluorescence recovery after photobleaching

PCNA: proliferating cell nuclear antigen

SSBR: single strand break repair

SSBs: single strand breaks

XRCC1: X-ray cross complementing factor 1

## Competing interests

The author(s) declares that there are no competing interests.

## Authors' contributions

OM designed and performed the experiments, analyzed the data and participated in writing the manuscript. HL participated in experimental design and writing the manuscript. All authors read and approved the final manuscript.

## Supplementary Material

Additional file 1 Laser microirradiation generates different types of DNA damage. Description: The data provided shows that laser microirradiation with a 405 nm laser generates different types of DNA damage, including SSBs and DSBs. Click here for file

Additional file 2 Mobility of XRCC1 and PCNA and their respective binding partners DNA Ligase III and I at DNA damage sites. Description: The data provided indicates that XRCC1 and its binding partner DNA Ligase III show similar turnover rates at DNA damage sites, while the mobility of PCNA and its binding partner DNA Ligase I differ dramatically. Click here for file
