# Supplementary material for: XRCC1 and PCNA are loading platforms with distinct kinetic properties and different capacities to respond to multiple DNA lesions
Source: BMC Mol Biol. 2007 Sep 19;8:81. doi: 10.1186/1471-2199-8-81 (PMC2039748; doi:10.1186/1471-2199-8-81)
Supplement: Additional file 1 — Laser microirradiation generates different types of DNA damage. Description: The data provided shows that laser microirradiation with a 405 nm laser generates different types of DNA damage, including SSBs and DSBs. [file 1471-2199-8-81-S1.doc]

**
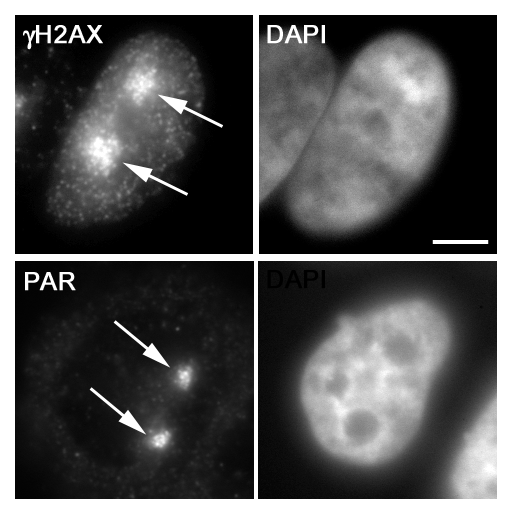
**

**Supplementary Figure 1**

Laser microirradiation generates different types of DNA damage. Widefield fluorescence images of Hela cells are shown. Fixation and immunostaining was performed ~5 min after laser microirradiation. Arrows mark sites of irradiation. Laser microirradiation results in local generation of DSBs (A) and SSBs (B) detected by antibodies against γ-H2AX and PAR, respectively. Scale bar, 5 µm.
