# Supplementary material for: XRCC1 and PCNA are loading platforms with distinct kinetic properties and different capacities to respond to multiple DNA lesions
Source: BMC Mol Biol. 2007 Sep 19;8:81. doi: 10.1186/1471-2199-8-81 (PMC2039748; doi:10.1186/1471-2199-8-81)
Supplement: Additional file 2 — Mobility of XRCC1 and PCNA and their respective binding partners DNA Ligase III and I at DNA damage sites. Description: The data provided indicates that XRCC1 and its binding partner DNA Ligase III show similar turnover rates at DNA damage sites, while the mobility of PCNA and its binding partner DNA Ligase I differ dramatically. [file 1471-2199-8-81-S2.doc]

**
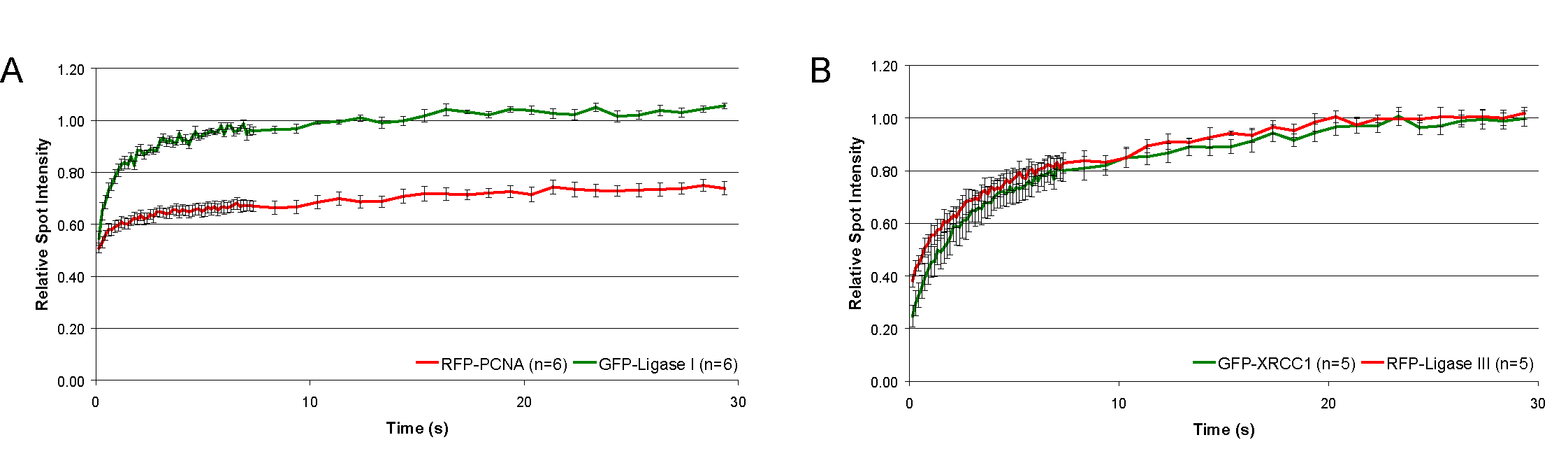
**

**Supplementary Figure 2**

Mobility of XRCC1 and PCNA and their respective binding partners DNA Ligase III and I at DNA damage sites. Two separate subnuclear spots of transiently transfected Hela cells were microirradiated and the mobility of accumulated fluorescent fusion proteins was determined as described in Figure 3. FRAP data from at least 5 different experiments are shown as mean curves. Error bars represent the standard error of the mean.
